# A Hybrid Inverse Kinematics Framework for Biomimetic Redundancy Resolution in 7-DoF Humanoid Arms

**DOI:** 10.3390/biomimetics11060408

**Published:** 2026-06-09

**Authors:** Yapeng Shi, Zhen Chen, Ivan Mokiets, Songhao Piao, Teng Zhang, Lianzhao Zhang

**Affiliations:** 1 Faculty of Computing, Harbin Institute of Technology, Harbin 150001, China; 25s10350@stu.hit.edu.cn (Z.C.); 24sf03443@stu.hit.edu.cn (I.M.); piaosh@hit.edu.cn (S.P.); 2 National Key Laboratory of Smart Farm Technologies and Systems, Harbin Institute of Technology, Harbin 150001, China; 3 State Key Laboratory of Robotics and System, Harbin Institute of Technology, Harbin 150001, China; 24b908047@stu.hit.edu.cn (T.Z.); lianzhao@stu.hit.edu.cn (L.Z.)

**Keywords:** biomimetic robotics, kinematic redundancy, humanoid manipulators, data-driven modeling

## Abstract

Resolving the kinematic redundancy of 7-DoF humanoid arms to generate natural, human-like motions remains a fundamental challenge in biomimetic robotics. This paper presents a hybrid inverse kinematics (IK) framework that learns a pose-dependent redundancy parameter and integrates it into a differential IK solver. Specifically, we employ the stereographic Shoulder–Elbow–Wrist (SEW) angle as a well-conditioned geometric parameterization. This formulation transforms the algorithmic singularity into a unidirectional half-line, which can be oriented outside the typical reachable workspace. To specify the optimal configuration within the self-motion manifold, a motion dataset was collected by teleoperating a humanoid arm via an anthropomorphic wearable exoskeleton. This approach translates operator-specific postural preferences into the robot’s joint space. A lightweight neural network was then trained to learn the mapping from end-effector poses to these operator-specific SEW angles. By incorporating the predicted SEW angle as a dynamic secondary objective in the null space of the primary tracking task, the proposed framework enables natural redundancy resolution while preserving end-effector tracking accuracy. Both simulations and real-robot experiments were conducted to validate the approach. Results show that, compared to the average performance of static fixed-parameter strategies, the proposed method improves the Joint Configuration Quality Index (CQI) by 22.5% and reduces energy costs by 11.3%. Moreover, the sub-millisecond inference latency (0.44 ms) facilitates seamless integration into real-time control pipelines.

## 1. Introduction

The synthesis of natural, human-like motion is crucial for robotic systems to operate safely and intuitively in human-centric environments. Humanoid robots are recognized as versatile platforms for complex environments, requiring motion synthesis that aligns with behaviors predictable to human users [1,2]. Unlike task-specific manipulators, humanoid robots are designed to operate in environments originally structured for humans, requiring not only functional task execution but also motion behaviors that are intuitive, predictable, and socially acceptable to humans [3,4,5]. As robots are deployed closer to humans, the quality and naturalness of their motion become critical factors influencing safety, efficiency, and human trust.

To achieve these human-centric capabilities, most modern humanoid robots are equipped with 7-degree-of-freedom (DoF) arms that kinematically mirror the human arm, such as NASA’s Valkyrie and PAL Robotics’ TALOS [6,7]. This additional DoF introduces kinematic redundancy, allowing the arm to perform secondary objectives while executing primary tasks [8]. A prevalent paradigm for commanding these systems is teleoperation, where task-space commands from interfaces such as Virtual Reality (VR) [9,10] or vision-based tracking [11] define the desired end-effector (EEF) pose. This decoupling between task-space commands and the robot’s kinematic structure effectively shifts the burden of configuration synthesis onto the inverse kinematics (IK) solver. For redundant manipulators, the IK problem admits infinitely many joint configurations for a single EEF pose, which necessitates a structured and interpretable representation of redundancy to guide configuration selection.

Conventional IK methods typically resolve redundancy by optimizing generic mathematical criteria, such as minimizing joint velocities or displacements [8]. Although these methods ensure task-space accuracy, they are often task-agnostic. Consequently, the generated configurations can appear perceptually unnatural and deviate from human postural strategies. This limitation stems from the absence of an explicit representation that captures operator-specific redundancy behaviors, which limits their ability to generate consistent and context-dependent configurations.

The Shoulder-Elbow-Wrist (SEW) angle, also known as the swivel angle, has emerged as a widely adopted geometric parameter for describing the postural configuration of 7-DoF arms, providing an intuitive interface between human motor strategies and robotic kinematics. Elias et al. [12] introduced a stereographic SEW formulation that transforms the traditional bidirectional singularity line into a unidirectional half-line. By strategically orienting this half-line outside the robot’s reachable workspace, this approach provides a well-conditioned geometric parameterization, effectively avoiding algorithmic singularities within the operational region. However, while the SEW angle robustly parameterizes the redundant space, determining its task-dependent value to mimic human coordination remains an open problem.

To address this limitation, we propose a hybrid approach that explicitly decouples redundancy resolution from the primary EEF tracking task. Specifically, a data-driven model learns a pose-dependent mapping from EEF targets to operator-specific SEW angles. By explicitly encoding operator-specific postural preferences, the neural predictor inherently restricts the self-motion manifold to biomimetically comfortable configurations. Ultimately, parameterizing redundancy within this low-dimensional geometric space establishes a robust interface between data-driven intent prediction and classical differential kinematic solvers.

Building on this theoretical insight, we present a hybrid IK framework for real-time redundancy resolution. To capture authentic operator-specific postural preferences, a motion dataset was first constructed via teleoperation using an anthropomorphic wearable exoskeleton. A lightweight multilayer perceptron (MLP) was then trained to efficiently map EEF poses to these learned SEW angles, satisfying the strict computational constraints of high-frequency control loops. Subsequently, the network-predicted SEW angle is incorporated as a dynamic secondary objective within a differential IK solver. This integration enables natural and consistent redundancy assignment while preserving precise EEF tracking accuracy. The main contributions of this work are as follows:

1. A hybrid IK framework that combines data-driven biomimetic behavior and classical numerical optimization. By decoupling redundancy resolution into a low-dimensional geometric space, the framework integrates learned operator-specific postural preferences into standard differential solvers while maintaining tracking accuracy.
2. An integrated learning paradigm for redundancy resolution, supported by a custom anthropomorphic exoskeleton for high-fidelity data acquisition. This formulation establishes a continuous SE(3) to $S^{1}$ mapping and demonstrates that explicit full-pose encoding is essential to resolve one-to-many kinematic ambiguity and capture operator-specific coordination patterns.
3. A real-time learning-based implementation validated on a 7-DoF humanoid arm, showing improvements in motion naturalness and efficiency, together with sub-millisecond inference latency for high-frequency control deployment.

The remainder of this paper is organized as follows: Section 2 reviews related work in redundancy resolution, focusing on the evolution of the SEW angle and the comparison between numerical and learning-based IK methods. Section 3 details the proposed hybrid IK framework, including the data acquisition process, the neural network architecture for SEW angle prediction, and the differential IK solver. Experimental results and performance evaluations are presented in Section 4, followed by conclusions and future work in Section 5.

## 2. Related Work

Solving the IK problem for redundant manipulators poses two fundamental challenges: (i) effectively parameterizing the infinite set of feasible joint configurations (i.e., the self-motion manifold), and (ii) selecting an optimal solution that satisfies task-specific or biomimetic criteria. Existing research addressing these challenges can be broadly categorized into numerical optimization methods, geometric parameterization approaches, and data-driven redundancy resolution strategies.

### 2.1. Numerical IK and Null-Space Control

Numerical methods form the foundation of modern IK solvers due to their generality and high precision. At the position level, IK is typically formulated as a nonlinear optimization problem and solved iteratively using Newton-type or gradient-based methods [13,14]. While effective, these approaches are often sensitive to initialization and computationally demanding [8]. Recent work has revisited redundancy resolution from a global perspective; for example, Hauser [15] proposed constructing continuous mappings over the self-motion manifold to achieve globally consistent solutions. However, such approaches remain primarily geometry-driven and do not explicitly account for human motion preferences.

At the velocity level, differential IK based on the Jacobian pseudoinverse remains the dominant paradigm for real-time control. Redundancy is typically resolved by projecting secondary objectives into the null-space of the Jacobian matrix [8,16]. Classical objectives include joint limit avoidance, singularity avoidance, and manipulability maximization [17,18].

Recent advances have extended velocity-level IK along several structured directions. One line of work focuses on improving physical consistency, through dynamically consistent or weighted Jacobian inverses, enabling more principled motion and force behaviors [19]. Another line integrates Jacobian-based local updates with global optimization or metaheuristic strategies to enhance robustness under constraints [20]. More recently, learning-assisted and model-free approaches have been explored to estimate or adapt the Jacobian online, allowing for increased flexibility in uncertain or partially modeled systems [21]. These developments align with broader advances in optimization-based and adaptive control frameworks for redundant robotic systems.

Despite these methodological advances, velocity-level IK methods share two fundamental limitations. First, mathematically, they rely on local differential representations and do not explicitly capture the global structure of the self-motion manifold. Consequently, they may lead to undesirable behaviors such as non-cyclic joint evolution for closed-loop EEF trajectories [22] and instability near singular configurations [23]. Second, behaviorally, their secondary objectives remain mathematically or physically driven rather than being grounded in empirical human demonstrations. Because these objectives are largely heuristic and task-agnostic, they fail to reproduce the context-dependent coordination patterns observed in human motion. As a result, the generated configurations are often kinematically feasible but perceptually unnatural.

### 2.2. Geometric Parameterization of the Self-Motion Manifold

To address the limitations of local redundancy control, explicit geometric parameterizations of the self-motion manifold have been proposed to provide a more global and interpretable representation. Recent studies have explored topological analyses of self-motion manifolds to ensure continuity and global consistency in redundancy resolution [24]. Among various representations, the SEW angle has emerged as a widely adopted geometric descriptor [25]. It encodes the orientation of the arm plane defined by the shoulder, elbow, and wrist, offering an intuitive and physically meaningful representation of arm posture [26]. This property ensures predictable and cyclic motion generation, leading to its widespread adoption in domains ranging from industrial manufacturing and teleoperation to service robotics [27,28,29,30].

While geometrically intuitive, the performance of the SEW angle framework heavily depends on its mathematical formulation. While topological constraints dictate that algorithmic singularities are unavoidable for any redundancy parameterization, traditional definitions based on a fixed reference vector suffer from a bidirectional singularity line that often intersects the usable workspace. To address this limitation, Elias et al. [12] introduced the stereographic SEW angle, which transforms this algorithmic singularity into a unidirectional half-line. By orienting this half-line toward an unreachable region (e.g., the robot’s base), algorithmic singularities are effectively excluded from the functional workspace. Nevertheless, while such kinematic representations successfully circumvent algorithmic singularities, they still leave the self-motion manifold underdetermined. Specifically, they lack an explicit mechanism to select a unique, biomimetic configuration. Our work addresses this limitation by using a data-driven model to navigate this singularity-free geometric space, so that the chosen redundancy resolution aligns with operator-specific postural preferences.

### 2.3. Data-Driven Redundancy Resolution

To actively generate biomimetic postures, recent research has increasingly turned to data-driven approaches, such as utilizing wearable demonstrations to transfer human-like manipulation skills to humanoid systems [31]. While foundational machine learning attempts sought to directly approximate the mapping from EEF poses to joint angles, modern deep learning methods have progressively shifted toward probabilistic and structured generation to address the inherent ill-posedness of redundant kinematics. To overcome the theoretical limitations of simple regression models, which often average distinct multimodal solutions into invalid intermediate postures, researchers have developed more expressive generative and hierarchical architectures. For instance, Bensadoun et al. [32] proposed IKNet, a hypernetwork-based framework that sequentially samples valid joint distributions conditioned on preceding joints, effectively resolving the one-to-many mapping problem. Similarly, Ho and King [33] developed a framework leveraging variational autoencoders (VAEs) to probabilistically navigate the redundant solution space, while Demby’s et al. [34] introduced a learning-by-example paradigm that incorporates prior joint-pose reference pairs to improve direct joint regression accuracy for complex serial robots.

Although these end-to-end architectures demonstrate the theoretical feasibility of learning complex kinematics, they suffer from two key limitations that hinder their deployment in high-frequency control: (i) they struggle to consistently guarantee the strict millimeter-level numerical precision required for physical robotic manipulation without subsequent computationally expensive numerical refinement, and (ii) modeling the multimodal solution space through complex generative architectures (e.g., sequential sampling or probabilistic inference) may incur increased and unpredictable inference latency, complicating real-time, deterministic control deployment [35].

Furthermore, optimization-based frameworks such as RelaxedIK [36] have provided significant advancements by framing the IK problem as a real-time multi-objective optimization task. These frameworks effectively navigate the redundant solution space by balancing task-space tracking accuracy with secondary objectives like joint-limit avoidance and manipulability. A complementary paradigm is to decouple redundancy resolution from the primary tracking task by learning a low-dimensional redundancy parameter, which can then be incorporated as a structured secondary objective in a standard differential IK solver.

In summary, current literature reveals a persistent trade-off among kinematic precision, biomimesis, and structural interpretability. Numerical methods provide high precision but typically rely on rigid heuristics; data-driven end-to-end models capture human motion preferences but often suffer from architectural opacity or inference latency; and optimization-based frameworks like RelaxedIK offer effective real-time retargeting through iterative multi-objective optimization. Fundamentally, most existing methods either operate in high-dimensional joint spaces or rely on abstract representations, limiting interpretability and controllability.

Rather than introducing a new optimization algorithm, this work takes a complementary perspective by focusing on the representation and parameterization of redundancy. Specifically, we propose to resolve redundancy within a low-dimensional, geometrically interpretable space, where operator-specific postural patterns can be learned and integrated in a structured manner. To this end, we develop a hybrid framework that decouples task-space tracking from redundancy resolution and learns the singularity-free stereographic SEW angle as a data-driven redundancy descriptor. This formulation allows the proposed method to remain fully compatible with standard differential IK solvers, while augmenting them with learned, operator-consistent null-space behaviors. In this way, the framework bridges the gap between analytical kinematic precision and data-driven postural naturalness without altering the underlying IK optimization paradigm.

## 3. Methodology

### 3.1. Problem Formulation

Given a desired EEF pose X∈SE(3), we adopt its numerical representation $x\in R^{7}$, consisting of the Cartesian position and a unit quaternion orientation. The 7-dimensional representation is used for learning, whereas the differential IK solver operates in a 6-dimensional spatial velocity space. The goal is to determine a corresponding joint configuration $q\in R^{7}$ that generates natural and human-like arm postures.

Due to kinematic redundancy, infinitely many joint configurations can achieve the same EEF pose. To explicitly resolve this redundancy, we introduce the stereographic SEW angle ψ (hereafter referred to simply as the SEW angle) as a low-dimensional geometric representation of the redundant configuration. Geometrically, ψ describes the rotation of the arm plane (spanned by the shoulder, elbow, and wrist joint centers) about the virtual axis connecting the shoulder and wrist, computed relative to a stereographic reference vector following [12]. The redundancy resolution problem is then formulated as learning a continuous mapping $M:SE\left(3\right)\to S^{1}$, expressed as:

(1) ψ=f(x),

where SE(3) is represented using a unit quaternion with sign ambiguity handled implicitly. f(·) captures operator-specific redundancy behaviors by mapping EEF poses to the corresponding preferred arm-plane orientation. Although multiple joint configurations q can correspond to the same operational pose x, resulting in different ψ values, the learned deterministic function f(x) approximates a consistent, statistically dominant redundancy preference (i.e., a representative solution within the distribution of demonstrated human motions) conditioned on the dataset distribution. This formulation implicitly assumes a single-valued regression model, where variability in human demonstrations is resolved through statistical consistency rather than computationally expensive multi-modal modeling. The predicted SEW angle is subsequently incorporated into a differential IK framework as a geometric null-space objective to drive the system toward a feasible joint configuration consistent with the predicted redundancy preference.

A key advantage of the proposed framework lies in its robustness against singularities. Geometrically, the stereographic SEW formulation compresses the traditional bidirectional singularity into a unidirectional half-line. By orienting this half-line toward the robot base (an unreachable region), algorithmic singularities are shifted outside the typical operational workspace under the chosen configuration.

### 3.2. Teleoperation Interface and Kinematic Mapping

To approximate the redundancy mapping f(x), a high-fidelity human motion dataset was acquired using a custom-designed, unactuated anthropomorphic exoskeleton. The device serves as a passive measurement interface that captures the operator’s arm motion, with the measured joint states directly mapped to the humanoid arm for teleoperation. This design enables simultaneous motion acquisition and execution. The kinematic structure of the exoskeleton is designed to be compatible with the 7-DoF humanoid arm, reducing retargeting discrepancies.

**Figure 1:**
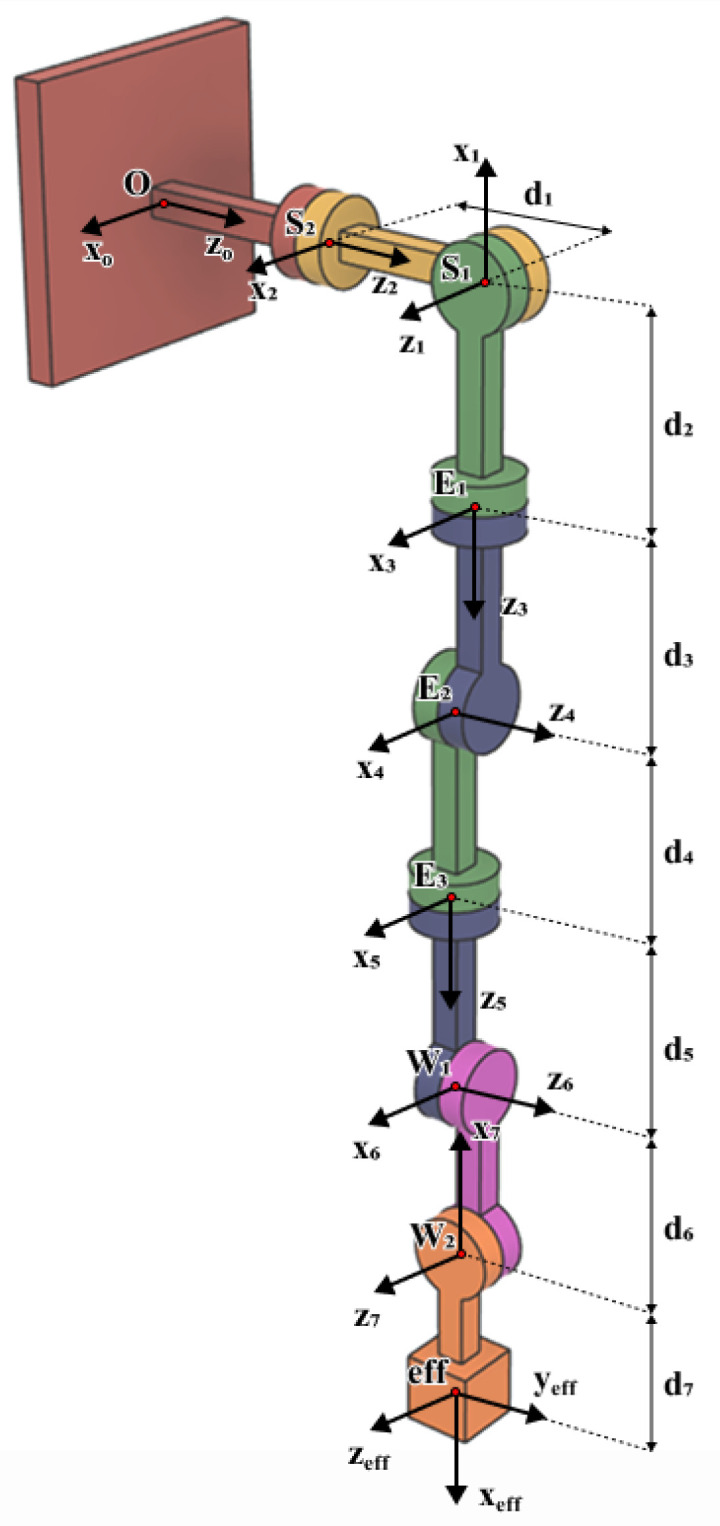
Kinematic diagram of the humanoid robot arm. The arm is represented as a seven-revolute-joint serial chain consisting of $S_{2}$, $S_{1}$, $E_{1}$, $E_{2}$, $E_{3}$, $W_{1}$, and $W_{2}$, followed by a fixed end-effector frame $E_{\mathrm{ff}}$. The coordinate frames and geometric parameters correspond to the DH parameters listed in Table 1.

**Figure 2:**
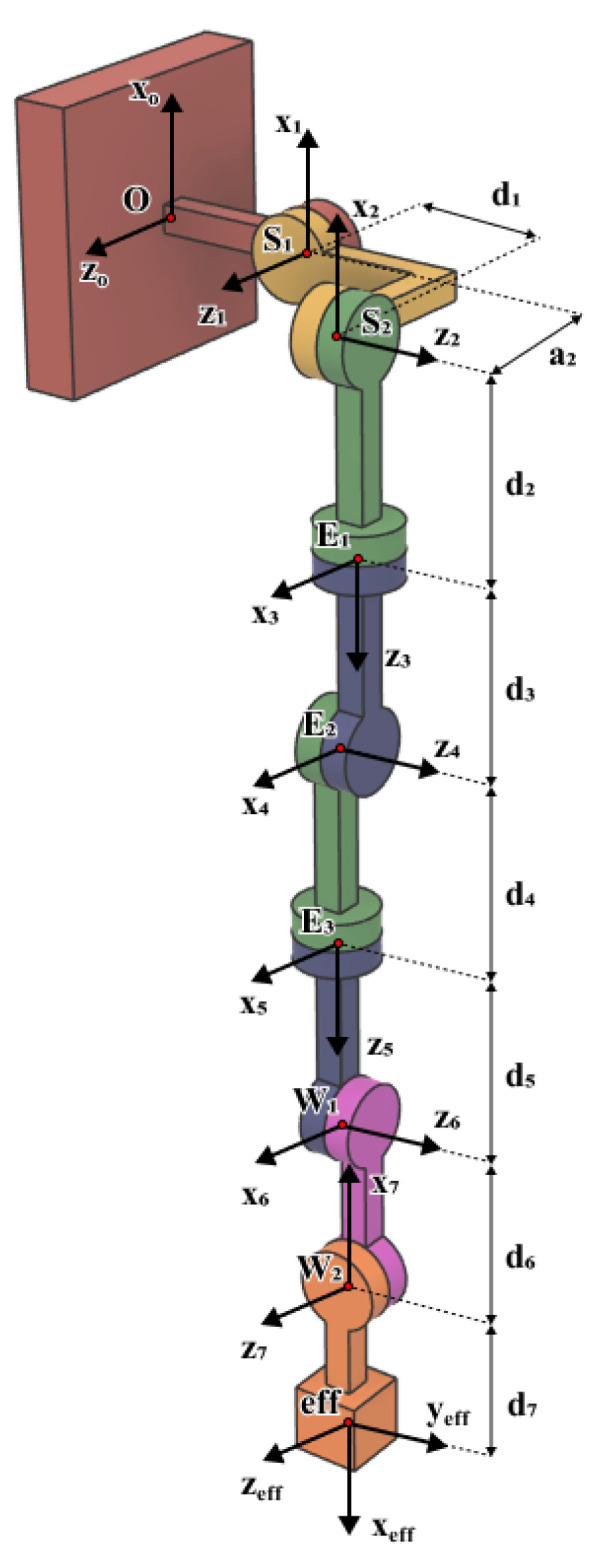
Kinematic diagram of the passive exoskeleton arm. The exoskeleton is represented as a seven-revolute-joint serial chain consisting of $S_{2}$, $S_{1}$, $E_{1}$, $E_{2}$, $E_{3}$, $W_{1}$, and $W_{2}$, followed by a fixed end-effector frame $E_{\mathrm{ff}}$. The coordinate frames and geometric parameters correspond to the DH parameters listed in Table 2.

The humanoid arm and the exoskeleton arm are described using the standard Denavit–Hartenberg (DH) convention, as shown in Figure 1 and Figure 2, respectively. The symbolic geometric parameters shown in the kinematic diagrams correspond to the measured physical dimensions of the mechanisms, and their numerical values are provided in the corresponding DH tables.

**Figure 3:**
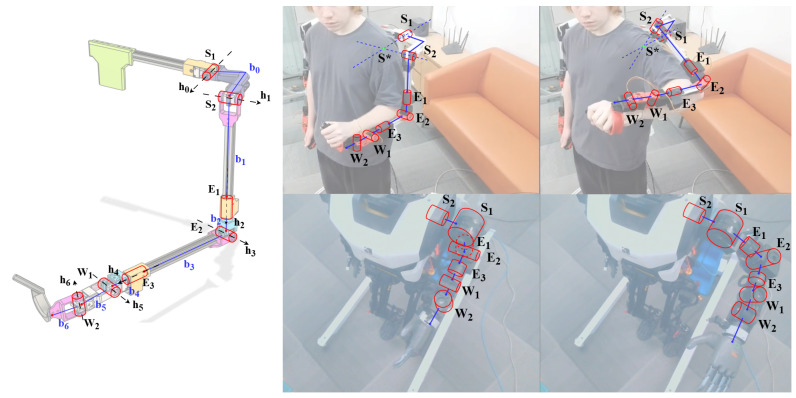
The teleoperation interface and kinematic mapping. The left panel shows the detailed kinematic structure of the exoskeleton, including joint modules ($S_{i}$, $E_{i}$, $W_{i}$), link vectors ($b_{i}$), and rotation axes ($h_{i}$). The remaining panels present representative teleoperation examples, illustrating the alignment between the operator’s arm posture, the exoskeleton kinematics, and the corresponding humanoid arm configuration.

Each arm of the exoskeleton consists of a serial chain of revolute joints aligned with the anatomical centers of the shoulder (*S*), elbow (*E*), and wrist (*W*), as illustrated in Figure 3. The kinematic structure is parameterized by link vectors $b_{i}\in R^{3}$ connecting consecutive joint centers, while revolute joint axes are denoted by unit vectors $h_{i}\in R^{3}$. The links are grouped into anatomical segments, including the upper arm ($b_{1}$–$b_{2}$) and the forearm-wrist chain ($b_{3}$–$b_{5}$). This parameterization defines a joint-space representation $q\in R^{7}$ that is directly compatible with the humanoid arm.

**Table 1:** Standard DH parameters of the humanoid robot arm. The row $E_{\mathrm{ff}}$ denotes the fixed end-effector frame and is not an actuated joint.

$J_{i}$	$a_{i}\;\left[\mathrm{mm}\right]$	$\alpha_{i}\;\left[\mathrm{rad}\right]$	$\theta_{i}\;\left[\mathrm{rad}\right]$	$d_{i}\;\left[\mathrm{mm}\right]$
$S_{2}$	0	0	$q_{S_{2}}^{r}$	80
$S_{1}$	0	$-\frac{\pi }{2}$	$q_{S_{1}}^{r}-\frac{\pi }{2}$	60
$E_{1}$	0	$+\frac{\pi }{2}$	$q_{E_{1}}^{r}+\frac{\pi }{2}$	105
$E_{2}$	0	$+\frac{\pi }{2}$	$q_{E_{2}}^{r}-\frac{\pi }{2}$	95
$E_{3}$	0	$-\frac{\pi }{2}$	$q_{E_{3}}^{r}$	40
$W_{1}$	0	$+\frac{\pi }{2}$	$q_{W_{1}}^{r}$	55
$W_{2}$	0	$-\frac{\pi }{2}$	$q_{W_{2}}^{r}-\frac{\pi }{2}$	85
$E_{\mathrm{ff}}$	0	0	0	180

**Table 2:** Standard DH parameters of the exoskeleton arm. The link dimensions are determined by the operator’s arm anthropometry and are independent of the humanoid robot arm dimensions. The row $E_{\mathrm{ff}}$ denotes the fixed end-effector frame and is not an actuated joint.

$J_{i}$	$a_{i}\;\left[\mathrm{mm}\right]$	$\alpha_{i}\;\left[\mathrm{rad}\right]$	$\theta_{i}\;\left[\mathrm{rad}\right]$	$d_{i}\;\left[\mathrm{mm}\right]$
$S_{2}$	115	0	$q_{S_{2}}^{e}$	105
$S_{1}$	0	$+\frac{\pi }{2}$	$q_{S_{1}}^{e}$	230
$E_{1}$	0	$-\frac{\pi }{2}$	$q_{E_{1}}^{e}+\frac{\pi }{2}$	50
$E_{2}$	0	$+\frac{\pi }{2}$	$q_{E_{2}}^{e}$	165
$E_{3}$	0	$-\frac{\pi }{2}$	$q_{E_{3}}^{e}$	55
$W_{1}$	0	$+\frac{\pi }{2}$	$q_{W_{1}}^{e}$	75
$W_{2}$	0	$+\frac{\pi }{2}$	$q_{W_{2}}^{e}+\frac{\pi }{2}$	50
$E_{\mathrm{ff}}$	0	0	0	80

A key design feature lies in the shoulder joint arrangement. The first two shoulder joints of the exoskeleton ($S_{1}$, $S_{2}$) implement a roll–pitch sequence, whereas the humanoid arm adopts a pitch–roll configuration. This ordering difference is imposed by the posterior-lateral mounting of the exoskeleton, which constrains the feasible alignment of joint axes relative to the anatomical shoulder center. To maintain kinematic compatibility during large-range motion, the roll and pitch axes are arranged to remain orthogonal and to intersect near the anatomical shoulder center, thereby preserving the effective spherical kinematics of the shoulder. Although the exoskeleton shoulder yaw axis does not strictly intersect with the roll–pitch axes as in the humanoid arm, the overall alignment remains sufficient for teleoperation, as evidenced by the large workspace coverage and consistent arm postures shown in Figure 3. Consequently, the mapping between exoskeleton and robot joints remains direct: the measured roll and pitch angles are assigned to the corresponding robot joints without requiring a Cartesian-level transformation, because the change in rotation order primarily affects the intermediate orientation representation rather than the reachable workspace or the underlying spherical shoulder behavior.

To quantify the retargeting discrepancy introduced by the non-intersecting shoulder-yaw axis of the exoskeleton, we performed an additional shoulder-level kinematic analysis. Instead of comparing the full exoskeleton and robot geometries, which would mix the effects of different link lengths, joint offsets, and frame definitions, we isolated the influence of the shoulder-yaw offset itself. For each recorded joint configuration, two shoulder models were evaluated. The first model represents an ideal spherical shoulder, where the yaw, pitch, and roll axes intersect at a single point. The second model uses the same shoulder structure, but includes the measured translational offset of the exoskeleton shoulder-yaw axis. For both models, the shoulder, elbow, and wrist points were reconstructed, and the corresponding SEW angles were computed. Given the shoulder, elbow, and wrist positions S, E, and W, the shoulder–wrist direction is defined as

(2) $$u=\frac{W-S}{∥W-S∥}.$$

The SEW angle was computed by projecting a reference vector r and the elbow vector E−S onto the plane normal to u:

(3) $$\begin{array}{l} r_{⊥}=\frac{r-(r^{T}u)u}{∥r-(r^{T}u)u∥},\\ e_{⊥}=\frac{\left(E-S\right)-\left({\left(E-S\right)}^{T}u\right)u}{∥\left(E-S\right)-\left({\left(E-S\right)}^{T}u\right)u∥}. \end{array}$$

The SEW angle is then obtained as

(4) $$\psi =atan2\left(u^{T}\left(r_{⊥}\times e_{⊥}\right),r_{⊥}^{T}e_{⊥}\right).$$

For each sample *k*, the SEW-angle discrepancy caused by the shoulder-yaw offset was computed as

(5) $$\Delta \psi_{k}={\mathrm{wrap}}_{\left[-\pi ,\pi \right]}\left(\psi_{k}^{\mathrm{offset}}-\psi_{k}^{\mathrm{ideal}}\right).$$

The RMS retargeting discrepancy was then calculated as

(6) $${\mathrm{RMS}}_{\Delta \psi }=\sqrt{\frac{1}{N}\sum_{k=1}^{N}{\left(\Delta \psi_{k}\right)}^{2}}.$$

The results are summarized in Table 3. Over the recorded trajectory, the median SEW-angle discrepancy was 1.55°, and 95% of the samples remained below 3.16°. Four samples out of 964 showed large discontinuities, which occurred near numerically unstable SEW configurations where the arm plane becomes poorly conditioned. After excluding these four diagnostic outliers, the RMS retargeting discrepancy was 1.86°, with a maximum inlier error of 6.18°. These results indicate that the non-intersecting shoulder-yaw axis introduces only a small bounded discrepancy for the vast majority of recorded configurations.

During teleoperation, the measured exoskeleton joint angles are converted into robot joint commands using an explicit calibrated joint-space mapping. Let the measured exoskeleton joint vector and the commanded robot joint vector be defined as

(7) $$\begin{array}{cc} q_{e} & ={\left[q_{S_{1}}^{e},q_{S_{2}}^{e},q_{E_{1}}^{e},q_{E_{2}}^{e},q_{E_{3}}^{e},q_{W_{1}}^{e},q_{W_{2}}^{e}\right]}^{T},\\ q_{r} & ={\left[q_{S_{2}}^{r},q_{S_{1}}^{r},q_{E_{1}}^{r},q_{E_{2}}^{r},q_{E_{3}}^{r},q_{W_{1}}^{r},q_{W_{2}}^{r}\right]}^{T}. \end{array}$$

The commanded robot joint vector is computed as

(8) $$q_{r}={\mathrm{clip}}_{\left[q_{r,\min},\;q_{r,\max}\right]}\left(q_{r}^{0}+A\left(q_{e}-q_{e}^{0}\right)\right),$$

where $q_{e}^{0}$ and $q_{r}^{0}$ denote the calibrated initial exoskeleton posture and the corresponding robot reference posture, respectively. The function clip(·) enforces the robot joint limits obtained from the robot model. With the joint ordering defined above, the constant mapping matrix A is

(9) $$A=\left[\begin{array}{ccccccc} -1 & 0 & 0 & 0 & 0 & 0 & 0\\ 0 & 1 & 0 & 0 & 0 & 0 & 0\\ 0 & 0 & 1 & 0 & 0 & 0 & 0\\ 0 & 0 & 0 & 1 & 0 & 0 & 0\\ 0 & 0 & 0 & 0 & 1 & 0 & 0\\ 0 & 0 & 0 & 0 & 0 & 1 & 0\\ 0 & 0 & 0 & 0 & 0 & 0 & 1 \end{array}\right].$$

This mapping explicitly accounts for the different shoulder joint ordering between the exoskeleton and the humanoid arm: the exoskeleton joints $S_{1}$ and $S_{2}$ correspond to the robot joints $S_{2}$ and $S_{1}$, respectively. The negative coefficient accounts for the opposite positive rotation convention of the mapped shoulder axis, while the elbow and wrist joints are mapped one-to-one. For compactness, let $\Delta q_{j}^{e}=q_{j}^{e}-q_{j}^{e,0}$. The scalar joint-to-joint relations are summarized in Table 4.

All joint angles are converted to radians before command publication. The mapping in Equation (8) is applied at each control cycle after encoder calibration and before sending the command to the robot controller.

During teleoperation, the operator relies on continuous visual feedback of the robot state. As shown in Figure 3, the exoskeleton maintains close kinematic alignment with the operator’s arm across a wide range of motions, enabling consistent capture of coordinated shoulder–elbow–wrist configurations. Importantly, all recorded data correspond to the executed configurations of the humanoid arm rather than the raw exoskeleton motion. As a result, the dataset reflects a physically realizable mapping between human motion and robot kinematics under the constraints of the robotic platform. Consequently, the recorded SEW angles capture a robot-conditioned redundancy resolution strategy, representing consistent and feasible configurations within the robot’s self-motion manifold while preserving human-like coordination patterns.

Joint angles are measured using AS5600 12-bit magnetic encoders (ams-OSRAM AG, Premstaetten, Austria), which determine rotation angle by detecting the magnetic field orientation of a paired permanent magnet per joint. The single-turn resolution is 12-bit, corresponding to 4096 distinct positions per full revolution, yielding a theoretical angular resolution of 360°/4096≈0.0879°/LSB. All joints are sampled at 100 Hz in synchrony with the control loop. Each joint module undergoes zero-point calibration, full-range calibration, and nonlinearity correction at assembly, followed by a system-level alignment check with the arm in a natural hanging posture to ensure measurement consistency across the kinematic chain. The multi-channel joint state $q\in R^{7}$, together with gripper commands, are synchronized and streamed via the Robot Operating System (ROS) for downstream processing.

### 3.3. Data Collection and Processing

Based on this teleoperation interface, a task-oriented motion dataset was constructed from joint trajectories for training the redundancy prediction network. A human operator performed diverse manipulation tasks through the exoskeleton, including grasping, lifting, and spatial object relocation. It should be noted that all tasks were conducted within an uncluttered, obstacle-free workspace. Under such conditions, the operator’s redundancy resolution strategies were governed primarily by minimum-effort and ergonomic principles, rather than complex, multi-modal collision-avoidance maneuvers. During this process, the humanoid arm was simultaneously driven via joint-space mapping, resulting in 100 continuous trajectory sequences. The raw data consisted of humanoid arm joint configurations $q\in R^{7}$, covering a wide range of natural, human-like arm motions.

The data acquisition process involved 100 continuous manipulation sequences performed by a single human operator. Each sequence lasted approximately 50 s on average, allowing the operator to comfortably execute fluid, multi-step manipulation tasks. With the teleoperation system synchronized to the 100 Hz control loop, this process generated a raw dataset of roughly 500,000 spatial frames. The demonstrations were designed to cover the robot’s primary functional workspace, bounded by a Cartesian region of 0.30 m × 0.90 m × 0.30 m in the frontal manipulation zone.

However, continuous high-frequency recording inherently produces adjacent frames with extremely high temporal correlation. To maximize the dataset’s information density and prevent the neural network from overfitting to static or highly redundant motion segments, we uniformly downsampled the raw pool to extract N=100,000 high-quality, spatially distinct samples for the final training dataset. Furthermore, leveraging the robot’s kinematic symmetry, we applied bi-lateral data augmentation by mirroring the right-arm demonstrations, symmetrically doubling the spatial diversity of the training distribution.

As summarized in Table 5, given the relatively low-dimensional mapping from SE(3) to a single scalar SEW angle, a dataset of 100,000 downsampled samples provides dense coverage of the task-relevant portion of the operator-specific motion distribution. Rather than attempting to uniformly populate the entire high-dimensional SE(3) space, which is difficult to sample uniformly, this dataset continuously tracks the specific, ergonomic trajectories naturally adopted during functional tasks. This sampling strategy provides a representative empirical basis for training the subsequent redundancy prediction network.

To transform the raw joint trajectories into the specific input-output pairs required for model learning, a kinematic preprocessing pipeline was developed using the Drake robotics framework. To decouple intrinsic arm coordination from whole-body locomotion, all kinematic states were expressed relative to the robot’s torso frame. Specifically, the robot’s URDF model was instantiated within a MultibodyPlant, with the torso rigidly anchored to the world origin. This frame-normalization strategy ensures that the dataset captures the internal configuration of the 7-DoF arm, rendering the learned mapping invariant to global base motion.

Following normalization, the necessary EEF pose variables were derived from the recorded humanoid arm joint configurations. Forward kinematics was utilized to compute the relative spatial transform between the torso and the EEF, yielding the 7-dimensional EEF pose vector x. Concurrently, the corresponding scalar SEW angle ψ was computed from the same joint configuration based on the humanoid arm geometry, thereby providing a compact representation of the instantaneous redundancy state associated with the given EEF pose.

Although both x and ψ are deterministically derived from q, the empirical mapping from x to ψ is not strictly one-to-one in the collected dataset due to variability in human motion. Consequently, a learning-based approach was adopted to approximate a consistent mapping from EEF pose to operator-specific redundancy resolution, yielding the paired dataset $D={\left\{\left(x_{k},\psi_{k}\right)\right\}}_{k=1}^{N}$. This formulation enabled the model to capture the underlying statistical regularities of the operator’s redundancy behavior.

Finally, a rigorous data cleaning and partitioning strategy was applied. After filtering numerical outliers and sensor noise at the frame level, the dataset was partitioned to prevent temporal data leakage. Instead of a naive frame-wise random split, which inevitably introduces strong temporal correlations between training and validation sets, a strict trajectory-level split was adopted. Specifically, 80 intact sequences are randomly allocated for training, while the remaining 20 geometrically distinct sequences are reserved for validation. This strategy ensured that the network was evaluated on entirely unseen motion patterns, providing an unbiased and rigorous evaluation of its spatial generalization capabilities. This established the learning problem as a regression from task space to redundancy space.

### 3.4. SEW Angle Prediction Network

To resolve the redundancy of the 7-DoF humanoid arm, we employed a data-driven approach to predict the SEW angle ψ from the EEF pose x. The prediction model, termed *MotionMLP-BN*, learns a mapping from $x\in R^{7}$ to the corresponding redundancy parameter. The 7-dimensional input x consists of a 3D Cartesian position and a 4D unit quaternion. Incorporating the full spatial pose, rather than merely the 3D position, is geometrically essential to constrain the arm plane and resolve the inherent one-to-many kinematic ambiguity (as validated in Section 4.2). Furthermore, the quaternion representation was intentionally selected over Euler angles to provide a continuous and singularity-free mapping of SO(3), which is essential for stable neural network training and avoiding the gradient discontinuities inherent in gimbal-lock scenarios. Instead of directly regressing the angular value, which suffers from discontinuity at 2π, we adopted a unit-circle representation to ensure numerical continuity. Specifically, the network predicts a 2-dimensional vector $\hat{y}={\left[y_{c},y_{s}\right]}^{T}$, corresponding to the ${\left[\cos\psi ,\sin\psi \right]}^{T}$ components of the SEW angle, which improves training stability and avoids discontinuities in the loss landscape.

**Figure 4:**
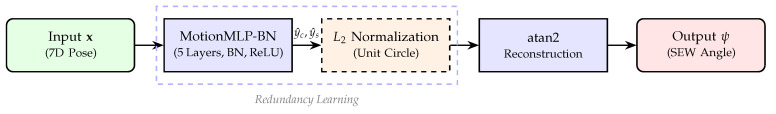
Architecture of the SEW prediction network. The model maps a 7D pose to the redundant SEW parameter space via a unit-circle transformation.

The architecture, illustrated in Figure 4, comprises five fully connected layers with hidden dimensions of 256 and 128. The hidden dimensions [256, 128] were selected to balance predictive capacity with computational efficiency. Increasing network depth or width provides marginal gains in RMSE at the cost of higher inference latency. Given the 100 Hz real-time control requirement of the humanoid arm, this lightweight architecture was chosen as the practical trade-off. Each linear layer (except the output layer) is followed by a Batch Normalization (BN) layer and a Rectified Linear Unit (ReLU) activation function to accelerate convergence and prevent internal covariate shift. To strictly constrain the output to the unit circle, a final $L_{2}$ normalization layer is applied to the raw 2D output of the preceding linear layer, denoted as $g\left(x\right)\in R^{2}$, such that $\hat{y}=g\left(x\right)/{(∥g\left(x\right)∥}_{2}+\epsilon )$, where $\epsilon =10^{-8}$ is a stability constant. During inference, the final SEW angle is reconstructed via $\psi_{pred}=atan2\left(y_{s},y_{c}\right)$.

The model was optimized by minimizing the Mean Squared Error (MSE) between the predicted 2D vector $\hat{y}$ and the ground truth labels $y^{*}={\left[\cos\psi^{*},\sin\psi^{*}\right]}^{T}$, where $\psi^{*}$ is the reference SEW angle derived from the humanoid arm kinematics. During the training phase, we employed the AdamW optimizer with an initial learning rate of 0.01 and a batch size of 256. A ReduceLROnPlateau scheduler was utilized to scale down the learning rate by a factor of 0.5 if the validation loss plateaued for 10 epochs, facilitating stable convergence. The network was trained for a maximum of 400 epochs, incorporating an early stopping mechanism with a patience of 150 epochs to prevent overfitting. To promote gradient stability, gradient clipping with a maximum norm of 0.9 was applied. The initial learning rate of 0.01 was adopted as a stable default for AdamW together with the ReduceLROnPlateau scheduler and was verified through the observed convergence behavior, rather than being treated as an exhaustively searched sensitivity variable. The gradient clipping norm of 0.9 was retained as a conservative guard against gradient spikes during unit-circle regression. Furthermore, Automatic Mixed Precision (AMP) was adopted to accelerate the training process while preserving numerical stability. To enhance the network’s robustness against real-world sensor noise and tracking jitter, a dynamic data augmentation strategy was implemented by injecting Gaussian noise $N(0,\sigma^{2})$ with a standard deviation of σ=0.005 into the 7D input features exclusively during training. The noise standard deviation σ=0.005 was chosen to be consistent with the physical resolution and empirical jitter profile of the magnetic encoders in the exoskeleton hardware, supporting generalization during real-world teleoperation.To provide an empirical guideline for hardware implementation, a targeted hyperparameter sensitivity analysis was conducted (Table 6). This analysis examines a practical range across three design dimensions: network architecture scaling, noise regularizer scales, and gradient clipping bounds.

First, regarding the network size, the dimensions around [256, 128] outline a practical operational window. While a tighter network [128, 64] exhibits a slight underfitting trend (1.18° RMSE), further expanding layers to [512, 256] offers diminishing tracking benefits (1.09° RMSE) while more than doubling the inference latency, which reduces real-time computational headroom. Second, for noise augmentation, while training without input-noise augmentation (σ=0) yields a lower RMSE in offline validation (0.86°), hardware deployment considerations suggest that omitting noise may reduce robustness to encoder jitter. Conversely, an excessive noise scale (σ=0.05) obscures the core kinematics mapping (2.05° RMSE). The scale of σ=0.005 forms a practical balance that accounts for encoder resolution and observed jitter while preserving predictable workspace mapping. Finally, the framework exhibits a relatively flat error landscape across the tested range of gradient clipping levels (0.1 to 2.0), with maximum variations remaining within approximately 0.05°. This small variance indicates that the system is not highly sensitive to clipping hyperparameter variations within the tested range. Because the initial learning rate was controlled by the ReduceLROnPlateau scheduler and verified through stable convergence behavior rather than included in the sensitivity sweep, the default training configuration is reported as LR = 0.01 with Clip = 0.9.

Although the general IK problem inherently admits multiple null-space solutions, the proposed framework employs a single-valued deterministic regression model. This formulation is motivated by both empirical observations and practical engineering considerations. First, under the uncluttered manipulation scenarios considered during data collection, the observed SEW angles for a given EEF pose exhibit low variability. This observation is consistent with commonly reported tendencies in human motion toward smooth and energetically efficient coordination [37,38]. Accordingly, the mapping from EEF pose to SEW angle can be approximated as a single-valued function, for which optimization via Mean Squared Error (MSE) provides a representative solution aligned with the dominant patterns in the dataset. Second, a deterministic mapping promotes spatial consistency during teleoperation, reducing unpredictable null-space variations and contributing to stable and predictable robot behavior.

### 3.5. Differential IK Solver

In a practical deployment scenario, such as VR-based teleoperation, the desired target EEF pose $x_{target}\in R^{7}$ is continuously streamed from human-driven task-space command interfaces. Upon receiving $x_{target}$, the aforementioned neural network immediately predicts the corresponding operator-specific SEW angle $\psi_{pred}$. The final stage of the framework then employs a standard velocity-level differential IK solver to integrate these signals and execute the motion.

The framework employs the classical Damped Least Squares (DLS) null-space projection as the execution backend, providing a deterministic and computationally efficient foundation for velocity-level kinematic control. Furthermore, the use of a standard differential solver highlights the modularity of the proposed approach, whereby the learned biomimetic objective ($\psi_{pred}$) can be directly incorporated without modifying the underlying numerical scheme.

For the 7-DoF humanoid arm, let $q\in R^{7}$ be the vector of joint angles. The relationship between the spatial velocity $\dot{\tilde{x}}\in R^{6}$ of the EEF and the joint velocities $\dot{q}$ is governed by the primary geometric Jacobian $J_{eef}\left(q\right)\in R^{6\times 7}$, such that $\dot{\tilde{x}}=J_{eef}\left(q\right)\dot{q}$. To ensure numerical stability near kinematic singularities, we employ the Damped Least Squares (DLS) method to compute the pseudo-inverse $J_{eef}^{†}$:

(10) $$J_{eef}^{†}=J_{eef}^{T}{\left(J_{eef}J_{eef}^{T}+\lambda^{2}I\right)}^{-1},$$

where λ is a damping coefficient used to bound joint velocities near singular configurations, and I is the identity matrix.

The joint velocity required for the primary tracking task is defined as ${\dot{q}}_{task}=J_{eef}^{†}K_{p}\Delta \tilde{x}$, where $\Delta \tilde{x}={\left[\Delta p^{T},\Delta \theta^{T}\right]}^{T}\in R^{6}$ represents the 6-dimensional task-space error. To bridge the dimensional gap between the 7-D learning space and the 6-D control space, the target pose $x_{target}$ is analytically mapped to a 6-D spatial error vector during each iteration. Specifically, the translational error $\Delta p\in R^{3}$ is computed as the direct Euclidean difference of the position components. While the network utilizes quaternions for predictive stability, the solver computes the rotational error $\Delta \theta \in R^{3}$ as an equivalent angle-axis representation. This ensures mathematical compatibility with the 6×7 geometric Jacobian, enabling efficient first-order velocity updates and consistent convergence toward the target pose. Here, $K_{p}$ is a positive definite gain matrix.

To resolve redundancy, the predicted SEW angle $\psi_{pred}$ is incorporated through a null-space projection. We define a secondary Jacobian $J_{\psi }=\partial \psi /\partial q\in R^{1\times 7}$, where ψ(q) is computed from the current arm configuration via the stereographic SEW formulation. The redundant joint velocity component ${\dot{q}}_{null}$ is computed by projecting the SEW optimization objective into the null-space of the primary Jacobian:

(11) $${\dot{q}}_{null}=\left(I-J_{eef}^{†}J_{eef}\right)J_{\psi }^{†}\left[k_{null}\Delta \psi \right],$$

where $k_{null}$ is the optimization gain, and the angular error $\Delta \psi =\mathrm{wrap}(\psi_{pred}-\psi_{curr})$ is strictly wrapped to the [−π,π] interval to ensure smooth and shortest-path joint adjustments. The term $\left(I-J_{eef}^{†}J_{eef}\right)$ serves as the null-space projection matrix. While utilizing the DLS-damped inverse $J_{eef}^{†}$ approximates the exact null-space projection, it effectively mitigates singularity-induced velocity spikes while maintaining negligible primary task interference.

The secondary Jacobian $J_{\psi }\in R^{1\times 7}$ relates joint velocities to the rate of change of the stereographic SEW angle, such that $\dot{\psi }=J_{\psi }\dot{q}$. Theoretically, $J_{\psi }$ can be decomposed into elbow (*E*) and wrist (*W*) components:

(12) $$J_{\psi }=J_{\psi ,E}J_{E}+J_{\psi ,W}J_{W},$$

where the partial derivatives with respect to the joint centers are:

(13) $$J_{\psi ,E}=\frac{{\left(e_{SW}^{\times }e_{CE}\right)}^{T}}{∥p_{CE}∥},$$

(14) $$J_{\psi ,W}=-e_{y}^{T}J_{f_{x}}-\frac{e_{SW}^{T}p_{SE}}{∥p_{SW}∥∥p_{CE}∥}{\left(e_{SW}^{\times }e_{CE}\right)}^{T}.$$

In the above formulations, $p_{S}$, $p_{E}$, and $p_{W}\in R^{3}$ denote the Cartesian positions of the shoulder, elbow, and wrist joint centers, respectively. $J_{E}$ and $J_{W}\in R^{3\times 7}$ represent the geometric Jacobians associated with the elbow and wrist positions. The unit vector along the shoulder-to-wrist axis is $e_{SW}=\left(p_{W}-p_{S}\right)/∥p_{W}-p_{S}∥$, $p_{CE}$ is the vector from the center of the SW axis to the elbow, and $e_{SW}^{\times }$ denotes the skew-symmetric matrix used for the cross-product operation. The vector $e_{y}$ and the Jacobian component $J_{f_{x}}$ follow the stereographic coordinate definitions in [12].

While the analytical formulation in Equations (12)–(14) provides an exact reference, the primary focus of this work is the hybrid integration of data-driven priors into standard IK pipelines. A numerical finite-difference approximation is intentionally utilized in the implementation to maintain modularity and generalizability across different robot embodiments without requiring re-derivation of geometric gradients. As shown in our validation study, this approximation provides acceptable accuracy and latency for the tested workspace and control setting, justifying it as a practical choice for real-time biomimetic control.

The total joint velocity $\dot{q}={\dot{q}}_{task}+{\dot{q}}_{null}$ is integrated to update the state: $q_{t+1}=\mathrm{clip}\left(q_{t}+\dot{q}\Delta t,q_{min},q_{max}\right)$. This iterative process continues until the translational and rotational tracking errors fall below their predefined thresholds ($\epsilon_{p}$ and $\epsilon_{R}$), as detailed in Algorithm 1.
**Algorithm 1** Differential IK Solver with SEW Constraint  1:**Input:** Target EEF pose $x_{target}$, Predicted SEW angle $\psi_{pred}$  2:**Output:** Optimized joint angles $q^{*}$  3:Initialize $q\leftarrow q_{current}$  4:**while** (${∥\Delta p∥}_{2}>\epsilon_{p}$ **or** ${∥\Delta \theta ∥}_{2}>\epsilon_{R}$) **and** iter<max_iter **do**  5:   $x_{curr},\psi_{curr}\leftarrow$ ForwardKinematics(q)  6:   $\Delta \tilde{x}\leftarrow$ ComputeSpatialPoseError($x_{target},x_{curr}$)  7:   $J_{eef}\leftarrow$ CalculateGeometricJacobian(q)  8:   $J_{\psi }\leftarrow$ CalculateSEWJacobian(q) {via Finite Difference}  9:   ${\dot{q}}_{task}\leftarrow J_{eef}^{†}K_{p}\Delta \tilde{x}$10:   Δψ← WrapAngle($\psi_{pred}-\psi_{curr}$)11:   ${\dot{q}}_{null}\leftarrow \left(I-J_{eef}^{†}J_{eef}\right)J_{\psi }^{†}\left[k_{null}\Delta \psi \right]$12:   $q\leftarrow \mathrm{clip}(q+\left({\dot{q}}_{task}+{\dot{q}}_{null}\right)\Delta t,q_{min},q_{max})$13:   iter←iter+114:**end while**15:**return** $q^{*}=q$

Although Algorithm 1 is structured with an iterative loop for spatial tracking, running it to full position-level convergence at every time step can induce unpredictable latency spikes, violating strict real-time control constraints. Therefore, in our dynamic teleoperation deployment, the solver is explicitly configured to execute only a strictly bounded number of iterations per control cycle. In a high-frequency control loop where the target EEF pose $x_{target}$ updates continuously, the solver uses the joint configuration from the immediate previous step ($q_{current}$) as a warm start. Under this truncated execution scheme, the algorithm effectively behaves as a high-frequency, single-step differential driver rather than a blocking position-level solver. This design keeps the per-cycle computation bounded and compatible with real-time control while ensuring that the system dynamically tracks the target manifold.

## 4. Experimental Results

This section systematically evaluates the proposed framework through four complementary experiments. We first benchmark neural architectures to identify a practical trade-off between predictive accuracy and real-time efficiency. We then conduct an ablation study to analyze input feature sensitivity and its role in capturing operator-specific postural coordination. Next, the manifold-guided approach is compared against conventional numerical and fixed-parameter solvers. Finally, a real-world deployment on a physical humanoid arm evaluates the framework’s robustness in a dynamic manipulation task.

### 4.1. Architecture Selection for SEW Prediction

To identify a suitable backbone architecture for real-time SEW prediction, we benchmarked five neural architectures, including a Tiny-MLP, the proposed MotionMLP-BN, two configurations of ResNet (Light/Deep), and a Transformer encoder. All models were trained under identical settings and evaluated on a held-out test set. Performance was measured using RMSE and inference latency on an NVIDIA 3060 GPU with a batch size of 1.

**Figure 5:**
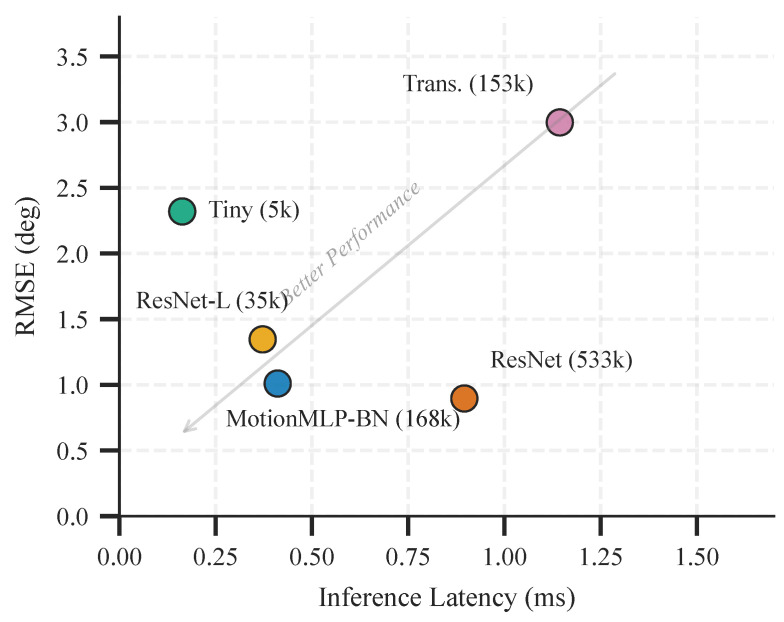
Trade-off analysis between predictive accuracy (RMSE) and inference latency. The parameter count for each model is shown in parentheses. Note that the proposed MotionMLP-BN and the ResNet-Light configurations offer the most viable balances for high-frequency control.

The quantitative results, summarized in Table 7 and visualized in Figure 5, reveal a clear trade-off between predictive precision and computational efficiency. While the ResNet (Deep) model established the accuracy ceiling with the lowest RMSE of 0.889°, its 0.936 ms inference latency introduces excessive computational overhead. Because the neural network functions as a pre-computation stage for the differential IK solver, minimizing its latency is critical to preserving computational headroom and maintaining a high-frequency control loop. For highly resource-constrained platforms, the ResNet (Light) configuration presented a compelling alternative, achieving a competitive RMSE of 1.346° using only 35k parameters. Conversely, the Transformer architecture underperformed, likely because the self-attention mechanism is overly complex for the low-dimensional mapping inherent to this specific task.

Balancing these extremes, the proposed MotionMLP-BN emerges as a well-balanced architecture for this framework. Compared to the lightweight ResNet, it substantially improved angular precision (RMSE 1.130° vs. 1.346°), which helps reduce postural jitter and supports smooth, human-like motion. At the same time, it kept the inference overhead to 0.44ms. This low computational overhead accounts for only a marginal fraction of the subsequent numerical optimization phase, indicating that the data-driven guidance does not substantially affect the high-frequency control loop. This balance supports the selection of MotionMLP-BN as the primary architecture for all subsequent analyses and real-world deployments.

### 4.2. Effect of Input Representation

The stereographic SEW angle is geometrically defined by the spatial orientation of the triangle formed by the shoulder, elbow, and wrist centers, effectively characterizing the orientation of the arm plane. While the 3D position of the EEF anchors the distal wrist location, it provides no constraint on the EEF orientation. From a kinematic perspective, this leads to different levels of ambiguity in redundancy resolution. For a 7-DoF humanoid arm, a full 6-DoF EEF pose constrains the system to a one-dimensional self-motion manifold. This strict constraint makes the SEW angle a well-defined, low-dimensional geometric representation of redundancy. In contrast, providing only a 3D position expands the feasible joint configurations to a higher-dimensional set. Consequently, multiple distinct arm postures can correspond to the exact same spatial point, resulting in a wide spectrum of valid SEW values for a single input. This one-to-many relationship renders the mapping from position to SEW inherently multi-valued, posing a significant challenge for deterministic learning-based approaches.

To systematically evaluate the impact of input representation, we constructed two distinct network configurations based on the proposed MotionMLP-BN. Both models shared identical architectures but differed exclusively in their input spaces: a **Position-Only** model with 3D Cartesian input (x,y,z), and a **Full-Pose** model using the complete 7D representation $\left(x,y,z,q_{w},q_{x},q_{y},q_{z}\right)$. Each model was trained independently from scratch, thereby constituting two distinct regression problems and enabling an unbiased assessment of how input completeness affects redundancy modeling.

**Figure 6:**
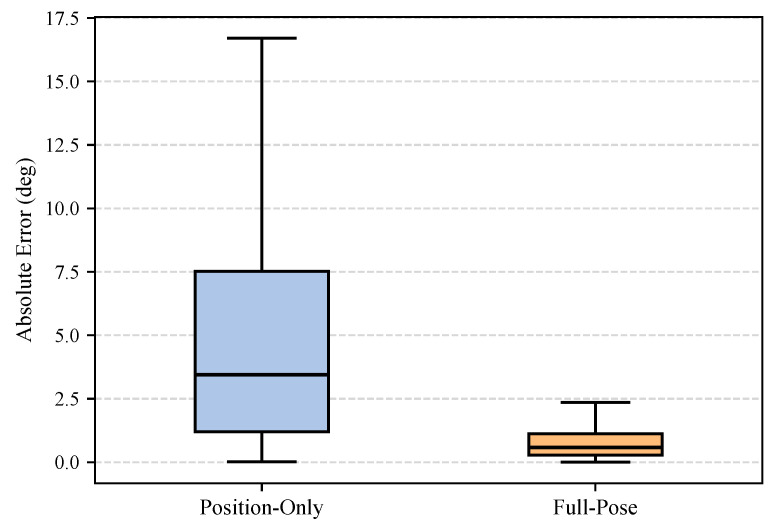
Error distribution comparison between **Position-Only** and **Full-Pose** models. The high variance in the **Position-Only** model reveals the geometric ambiguity caused by the absence of orientation context, which is critical for capturing operator-specific postural coordination.

As summarized in Table 8, incorporating orientation information reduced the RMSE from 7.13° to 1.13°, corresponding to an 84.2% improvement in predictive accuracy. Furthermore, the error distribution in Figure 6 shows that the **Position-Only** model exhibited extreme variance, indicating unstable predictions. This result supports the theoretical analysis: without explicit orientation constraints, the SEW angle cannot be reliably inferred, as the underlying mapping is ill-posed. Consequently, when presented with distinct human demonstrations corresponding to the same spatial position, the **Position-Only** network tends to converge toward a mathematical average of these configurations, leading to substantial predictive ambiguity.

By contrast, the **Full-Pose** representation provides sufficient geometric context to constrain the arm plane, effectively reducing the ambiguity of the self-motion manifold. The network is thus able to learn consistent mappings that capture coordinated relationships between EEF pose and elbow configuration. This behavior aligns with observed patterns in human motion, where arm posture is jointly influenced by both hand position and orientation, enabling efficient and natural movement strategies.

### 4.3. Comparison of Null-Space Formulations for Redundancy Resolution

To evaluate the effectiveness of the proposed redundancy resolution, we conducted a controlled comparison under a unified, classical differential IK framework. By keeping the underlying numerical DLS solver completely standard and constant across all baselines, we isolated the kinematic impact of the null-space objective itself. Specifically, three representative strategies were considered: (i) **Pure DLS**, where redundancy remains unconstrained, yielding the classical minimum-velocity-norm solution; (ii) **Fixed SEW**, where a constant SEW angle (−90°, −135°, or −180°) is imposed as a static null-space constraint; and (iii) the **Proposed Method**, where the SEW angle is predicted from the EEF pose and enforced as a context-dependent objective. The evaluation was conducted using three standard kinematic metrics: the Manipulability Index ($w=\sqrt{\det(J_{eef}J_{eef}^{T})}$), the Joint Configuration Quality Index (CQI) [39], and Motion Energy ($E={\sum ∥\Delta ,q∥}^{2}$).

Quantitative results over 100 continuous motion sequences are reported in Table 9. As expected, the Pure DLS strategy achieved the lowest motion energy (E=5.82) due to its minimum-norm formulation. However, this efficiency came at the severe expense of postural quality, reflected by a low joint configuration quality index (CQI=0.4215). Introducing a fixed SEW constraint improved posture in certain regions of the workspace. Nevertheless, its static nature resulted in inconsistent global performance, since one single angle could not adapt to all spatial configurations.

The absolute values of the Manipulability Index *w* (0.0038–0.0044) in Table 9 are reported using the adopted geometric-Jacobian convention. This $10^{-3}$ magnitude is consistent with calculating the product of six singular values for a manipulator of this scale, where the translational components are constrained by the arm’s total length (approximately 0.4 m). The estimated maximum manipulability over the sampled reachable workspace under the same convention is approximately 0.0065. Thus, the proposed method’s mean value of 0.0041 corresponds to 63.1% of the maximum and achieves a 7.9% improvement over the Pure DLS baseline (w=0.0038). Since these values are calculated analytically within a controlled simulation, they are not affected by sensor measurement noise and indicate a consistent kinematic trend in the tested trajectories, while simultaneously maintaining a high postural quality (CQI=0.5634).

In contrast, the proposed method achieved a favorable trade-off. Compared to the unconstrained Pure DLS, it maintained a high joint configuration quality index (CQI=0.5634), representing a 33.7% improvement, while incurring only a marginal 8.8% increase in motion energy (E=6.33). Although the fixed ψ=−135° strategy yielded a slightly higher mean CQI in this aggregate metric, its static parameter cannot adapt to different workspace regions. Furthermore, when compared to the average performance of the static Fixed SEW strategies, the proposed method not only improved the overall CQI by 22.5% but also reduced energy consumption by 11.3%. This demonstrates that the learned SEW mapping effectively regularizes the self-motion manifold, guiding the system toward operator-specific configurations without significantly compromising efficiency.

**Figure 7:**
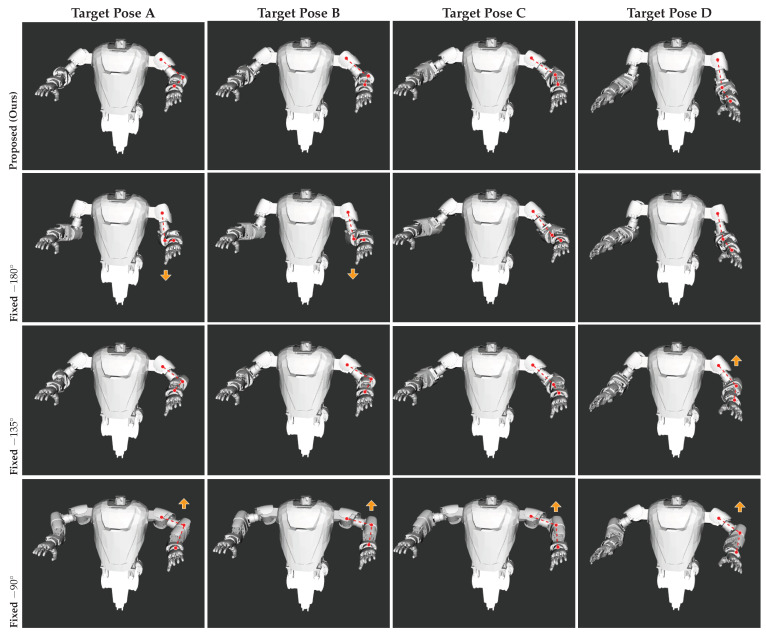
Visual comparison of redundancy resolution strategies. The **red dashed lines** and markers reconstruct the 3D arm skeleton (S-E-W linkage). **Yellow arrows** characterize suboptimal postural trends: **downward arrows** highlight elbow collapsing, while **upward arrows** denote excessive elevation. The **Top Row** shows our Proposed Model, which adaptively avoids these artifacts and maintains natural configurations across the entire workspace.

Qualitative comparisons in Figure 7 further highlight these differences. Note that the Pure DLS solver is omitted due to its fundamental lack of cyclic consistency, which leads to path-dependent elbow drifting. Among the static strategies, the fixed −180° strategy exhibited severe elbow collapse (indicated by downward arrows) in Poses A and B, significantly reducing torso clearance. Conversely, the fixed −90° strategy forced an unnaturally high shoulder elevation (upward arrows) across the evaluated poses. Even intermediate configurations failed under extreme poses, reflecting the inherent limitations of static redundancy priors. In contrast, the proposed method adapts continuously to task variations, producing smooth, consistent, and biomimetic arm configurations.

Overall, the results reveal a clear progression in redundancy resolution: evolving from unconstrained optimization (Pure DLS), to static geometric control (Fixed SEW), and ultimately to adaptive, learned null-space guidance. This progression highlights the necessity of modeling redundancy as a pose-dependent function in order to achieve both natural motion and robust kinematic performance.

### 4.4. Real-World Pick-and-Place Demonstration

To validate the practical performance of the proposed hybrid IK framework on physical hardware, we conducted a real-world pick-and-place experiment using a 7-DoF humanoid arm. The system operated at a control frequency of 100 Hz, generating a high-density dataset of 4000 synchronized sampling points over a 40-s continuous manipulation trial. Convergence thresholds for the execution were defined as $\epsilon_{p}=10^{-3}$ m for position and $\epsilon_{R}=10^{-3}$ rad for orientation.

**Figure 8:**
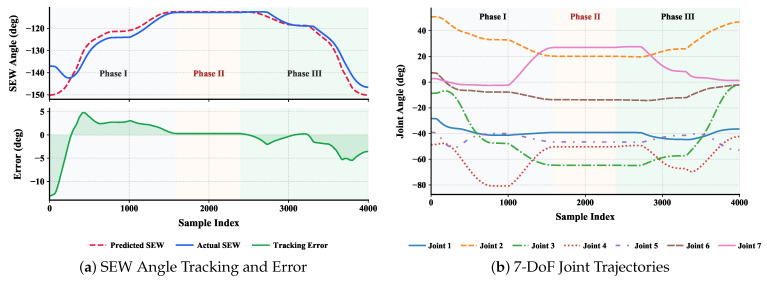
Kinematic performance analysis of the 40-s pick-and-place task (f=100 Hz, N=4000). (**a**) Tracking performance between the predicted and actual SEW angles, where the 100 Hz sampling reveals a consistent first-order tracking lag. (**b**) Corresponding 7-DoF joint trajectories, demonstrating $C^{1}$ continuity and the elimination of null-space drift during the stationary phase (Phase II).

The continuous manipulation sequence is structured into three fundamental operational phases: a dynamic approach towards the target object (Phase I), a stationary interval where the EEF remains strictly fixed to ensure a secure grasp (Phase II), and a dynamic return to the delivery zone (Phase III). The system’s control fidelity across the 4000 sampling points is quantitatively evaluated through dynamic redundancy tracking and joint-level execution stability, as illustrated in Figure 8. The high-density sampling reveals fine-grained tracking dynamics, demonstrating that the SEW angle transitions remain smooth without noticeable jitter even during rapid direction changes.

Regarding redundancy resolution accuracy, an initial SEW error of 13.1° was observed at the task’s onset, which stemmed from the spatial gap between the robot’s hardware idle pose and the learned manifold. The solver demonstrated robust error correction capability, rapidly driving the SEW error below $1^{{}^{\circ}}$ shortly after initialization. A deeper examination of the continuous tracking error ($\epsilon =\psi_{pred}-\psi_{curr}$) reveals a characteristic directional symmetry: a positive deviation during the approach (Phase I) and a negative deviation during the return (Phase III). This phenomenon is a direct manifestation of the velocity-level tracking lag inherent in the differential IK framework. Because redundancy resolution is governed by a proportional control law in the null-space (Equation (11)), it creates a first-order tracking system where the steady-state error is strictly coupled with the rate of change of the dynamic reference ($\epsilon \propto {\dot{\psi }}_{pred}$).

Throughout the 40-s trial, the representative 7-DoF joint trajectories maintained high-order smoothness, without observable joint-space discontinuities. Crucially, during the stationary interval (Phase II), the tracking error converged to near-zero (<0.5°), and the 7-DoF joint angles remained statically stable. The negligible null-space drift under stationary EEF constraints confirms the high numerical stability of the decoupled differential solver at a 100 Hz operational frequency.

**Figure 9:**
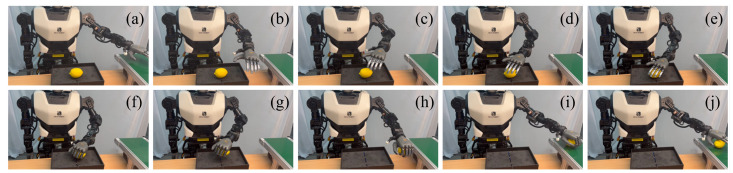
Snapshots of the physical pick-and-place manipulation. Subfigures (**a**–**j**) depict the continuous execution from the initial approach to the final delivery, illustrating the collision-free arm configurations and postural consistency achieved by the proposed hybrid framework.

The physical execution of this continuous manipulation cycle is captured through the ten sequential snapshots in Figure 9. By evaluating the framework across both moving and static task-space constraints, this phase-based design evaluates the system’s kinematic adaptability. The resulting visual sequence shows the fluid motion transitions and the consistent maintenance of biomimetic elbow configurations throughout the entire workspace, suggesting that the numerical stability of the hybrid solver contributes to reliable and natural robotic manipulation.

The Cartesian tracking fidelity during these physical maneuvers is quantified across the entire 40-s sequence. As summarized in Table 10, the proposed framework maintains a mean position error of 0.9794 mm and a mean orientation error of 0.0022 rad. Furthermore, the low standard deviation of the position error (0.0627 mm) indicates highly consistent spatial convergence throughout the dynamic trajectory. The 95th percentile (P95) position error remains below 0.9980 mm, indicating that the secondary SEW objective is effectively decoupled from the primary task space. This suggests that the dynamic redundancy resolution does not compromise the millimeter-level precision required for the tested manipulation task.

A timing and stability analysis of the proposed hybrid framework was performed on a high-performance workstation equipped with a 13th Gen Intel Core i9-13900K CPU (Intel Corporation, Santa Clara, CA, USA) and 32 GB RAM, running Ubuntu 20.04 LTS. As demonstrated in our architecture benchmarks, the neural SEW predictor achieved a sub-millisecond inference latency of 0.44 ms. Building upon this guidance, the differential IK solver was implemented in C++ and optimized within the Drake environment. Detailed latency and accuracy statistics over 4550 experimental cycles are summarized in Table 11. The solver achieved a mean total latency of 4.63 ms with a 95th percentile (P95) latency of 7.28 ms, indicating that the framework meets the 100 Hz control budget for most tested cycles. Notably, while the latency remains below 7.78 ms up to the 98th percentile (P98), a spike is observed at the 99th percentile (P99 = 20.41 ms). This localized long-tail distribution reflects the DLS safety mechanism: in rare edge cases approaching kinematic singularities, the damping limits aggressive velocities, causing the solver to use the full iteration budget. Furthermore, the numerical computation of $J_{\psi }$ via finite differences is efficient, requiring only 0.11 ms on average, which accounts for approximately 2.4% of the total solver time. A validation study shows that this numerical approximation achieves acceptable agreement with the analytical gradient, with a mean $L_{2}$ norm error of 0.291. The solver showed no observed instances of kinematic divergence or algorithmic failure across the sampled reachable workspace. Even when encountering the aforementioned edge cases that exhausted the iteration budget, the solver yielded bounded tracking errors rather than unstable joint behaviors. This execution behavior is partly attributed to the learned SEW objective, which guides the arm towards operator-specific configurations that tend to keep the arm away from mechanical joint limits, thereby improving operational consistency in real-time humanoid control pipelines.

### 4.5. Discussion

As previously noted, preliminary dry-runs with standard unconstrained solvers (e.g., Pure DLS) produced unfavorable behaviors on physical hardware, precluding a direct and safe dynamic hardware comparison. These behaviors are consistent with the sim-to-real gap, which warrants a detailed theoretical discussion. For the proposed framework, the algorithmic pipeline is identical in simulation and hardware: the target EEF pose is mapped to a predicted SEW reference, which is then incorporated into the differential IK solver. Therefore, the sim-to-real issue mainly arises from hardware-side effects rather than from a change in the kinematic formulation itself. A practical challenge in deploying differential redundancy resolution algorithms from simulation to physical hardware is the inevitable presence of encoder quantization, calibration offsets, actuator tracking lag, joint friction, unmodeled motor dynamics, and sensor noise. In an ideal simulation, exact velocity-level differential mappings can ideally maintain a stationary null-space. However, on physical hardware, microscopic execution errors and noise accumulate over time through continuous numerical integration. For unconstrained solvers, these effects can accumulate freely within the unconstrained null-space and can appear as unpredictable postural drift and undesirable postural variation in real-world tests.

The proposed hybrid scheme helps reduce the effect of this gap on redundancy drift by providing a pose-conditioned redundancy reference rather than relying on unconstrained null-space integration. By introducing the learned SEW predictor, the system establishes a continuous regularizing reference for the null-space objective. The MotionMLP-BN network predicts the desired biomimetic SEW angle based on the current Cartesian target, while the differential IK solver computes the current SEW angle from hardware joint feedback at each control cycle. In this sense, the learned predictor supplies the reference, and the null-space controller provides feedback regulation through the SEW tracking error. At every 10 ms control cycle, the analytical null-space projection guides the physical manipulator toward the learned biomimetic manifold. This feedback-regulated null-space objective attenuates accumulated integration errors and reduces the tendency toward unbounded null-space drift, thereby supporting more bounded postural behavior during teleoperation.

The present study centers on a controlled comparison among classical differential IK and fixed-parameter SEW strategies, whereas direct evaluation against end-to-end learning baselines such as IKNet [32] and VAE-based methods [33] remains an important direction for future investigation. For custom humanoid kinematics, these generative approaches typically require substantial architecture adaptation, hyperparameter tuning, and embodiment-specific training data. In addition, when Cartesian tracking accuracy is critical, end-to-end joint prediction methods may still require subsequent numerical correction, which can reduce their suitability for deterministic high-frequency control. In contrast, the proposed framework confines learning to the low-dimensional SEW subspace while preserving solver-based end-effector tracking. This design maintains the interpretability and numerical reliability of differential IK, while enabling operator-consistent redundancy resolution learned from demonstration. Accordingly, the present results should be interpreted as evidence that the hybrid formulation provides an effective balance among tracking accuracy, postural naturalness, and real-time feasibility under the tested single-operator, uncluttered setting. Future work will extend this formulation toward probabilistic or multi-modal null-space priors, together with broader validation in cluttered environments and across multiple operators.

## 5. Conclusions

This paper presents a hybrid inverse kinematics framework for resolving the redundancy of 7-DoF humanoid arms by combining demonstration-informed SEW prediction with differential task-space control. By learning a continuous mapping from the end-effector pose to an operator-specific stereographic SEW angle, the proposed method links mathematically rigorous end-effector tracking with more natural and task-consistent postural behavior.

The experimental results support the effectiveness of this formulation from three complementary perspectives. First, the ablation study demonstrates that full 7D pose information is important for capturing operator-specific postural coordination, reducing SEW prediction error by 84.2%. Second, the selected MotionMLP-BN achieves high predictive accuracy (1.13° RMSE) with low inference latency (0.44 ms), supporting deployment within high-frequency control pipelines. Third, relative to the average performance of traditional fixed-parameter strategies, the proposed dynamic redundancy resolution improves the overall CQI by 22.5% and reduces motion energy cost by 11.3%, while the hardware experiment further indicates the framework’s practical modularity and robustness under real robotic execution.

Although the present framework adopts a deterministic regression model and is validated under a single-operator, uncluttered setting, it establishes a clear and extensible foundation for data-informed redundancy resolution in humanoid manipulation. Future work will investigate probabilistic or multi-modal SEW priors to represent richer redundancy manifolds and to support more diverse task constraints, including obstacle-aware motion generation in less structured environments. A Supplementary Video (Video S1) provides a visual overview of the proposed workflow, including exoskeleton-based human-motion collection, pose-dependent SEW preference learning, and real-time humanoid-arm motion generation.

## Figures and Tables

**Table 3:** SEW-angle retargeting discrepancy caused by the non-intersecting shoulder-yaw axis.

Metric	Raw Statistics	Non-Degenerate Configurations
Number of samples	964	960
Mean absolute error (deg)	2.23	1.61
RMS error (deg)	9.90	1.86
Median error (deg)	1.55	–
95th percentile error (deg)	3.16	3.05
99th percentile error (deg)	5.26	3.81
Maximum error (deg)	156.68	6.18
Outlier samples, |Δψ|>30°	4	0

**Table 4:** Explicit joint-to-joint mapping from measured exoskeleton angles to commanded robot joint angles.

Exoskeleton Joint	Robot Joint	Mapping Relation
$S_{1}$	$S_{2}$	$q_{S_{2}}^{r}=q_{S_{2}}^{r,0}-\Delta q_{S_{1}}^{e}$
$S_{2}$	$S_{1}$	$q_{S_{1}}^{r}=q_{S_{1}}^{r,0}+\Delta q_{S_{2}}^{e}$
$E_{1}$	$E_{1}$	$q_{E_{1}}^{r}=q_{E_{1}}^{r,0}+\Delta q_{E_{1}}^{e}$
$E_{2}$	$E_{2}$	$q_{E_{2}}^{r}=q_{E_{2}}^{r,0}+\Delta q_{E_{2}}^{e}$
$E_{3}$	$E_{3}$	$q_{E_{3}}^{r}=q_{E_{3}}^{r,0}+\Delta q_{E_{3}}^{e}$
$W_{1}$	$W_{1}$	$q_{W_{1}}^{r}=q_{W_{1}}^{r,0}+\Delta q_{W_{1}}^{e}$
$W_{2}$	$W_{2}$	$q_{W_{2}}^{r}=q_{W_{2}}^{r,0}+\Delta q_{W_{2}}^{e}$

**Table 5:** Statistical Breakdown of the Motion Dataset.

Metric	Value
Total Demonstration Sequences	100
Average Duration per Sequence	≈50 s
Hardware Sampling Frequency	100 Hz
Total Raw Frames Generated	≈500,000
**Final Training Dataset Size (** ***N*** **)**	100,000
SEW Variance at Adjacent Poses ($\sigma_{\psi }$)	2.15°
Cartesian Workspace (X-Y-Z)	0.30 m × 0.90 m × 0.30 m

**Table 6:** Sensitivity analysis of MotionMLP-BN training hyperparameters.

Test Group	Hyperparameter	RMSE (°)	Latency (ms)
Model Size	[128, 64]	1.18	0.34
	[256, 128]	1.13	0.44
	[512, 256]	1.09	0.92
Noise σ	0	0.86	0.44
	0.005	1.13	0.44
	0.05	2.05	0.44
Grad Clip	0.1	1.08	0.44
	0.9	1.13	0.44
	2.0	1.09	0.44

**Table 7:** Performance Comparison of Candidate Neural Architectures. The gray background indicates the models selected as the optimal trade-offs for real-time deployment.

Model	RMSE (°)	Latency (ms)	Params (k)
Tiny-MLP	2.371	0.183	4.8
ResNet (Light)	1.346	0.372	35
MotionMLP-BN	1.130	0.437	168
ResNet (Deep)	0.889	0.936	533
Transformer	2.016	1.299	153

**Table 8:** Effect of Input Representation on SEW Prediction Performance. The gray background highlights the proposed configuration, and the upward arrow (↑) indicates the percentage of improvement.

Configuration	Input Dim	RMSE (°)	Max Err. (°)	Improv.
Position-Only	3	7.13	25.44	-
Full-Pose (Ours)	7	1.13	7.85	↑ 84.2%

**Table 9:** Quantitative Comparison of Kinematic Performance. The gray background separates different strategy categories, bold values indicate the best performance in each metric, and arrows (↑/↓) denote whether higher or lower values are preferred.

Strategy	Mean *w* (↑)	Mean CQI (↑)	Energy *E* (↓)
Pure DLS	0.0038	0.4215	**5.82**
Fixed $\psi =-90^{{}^{\circ}}$	0.0043	0.3915	7.74
Fixed $\psi =-135^{{}^{\circ}}$	0.0040	**0.5708**	6.88
Fixed $\psi =-180^{{}^{\circ}}$	**0.0044**	0.4177	6.80
**Proposed (Ours)**	0.0041	0.5634	6.33

**Table 10:** End-Effector Tracking Accuracy Statistics on Physical Hardware.

Error Metric	Mean	Std. Dev.	P95
Position Error (mm)	0.9794	0.0627	0.9980
Orientation Error (rad)	0.0022	0.0018	0.0045

**Table 11:** Detailed Performance and Accuracy Statistics (Evaluated on i9-13900K).

Metric	Mean	Std. Dev.	P90	P95	P98	P99
Total IK Solver Time (ms)	4.63	2.61	6.95	7.28	7.78	20.41
Single Jacobian (FD) Time (ms)	0.11	0.002	0.11	0.11	0.11	0.11
Jacobian $L_{2}$ Error (vs. Ana.)	0.291	0.078	0.389	0.412	0.458	0.525

## Data Availability

The data presented in this study are available on request from the corresponding author.

## Supplementary Materials

The following supporting information can be downloaded at: https://www.mdpi.com/article/10.3390/biomimetics11060408/s1, Video S1: Visual overview and experimental demonstration of the proposed hybrid inverse kinematics framework, including exoskeleton-based human-motion collection, pose-dependent SEW preference learning, and real-time humanoid-arm motion generation.
